# Intermediate phenotypes underlying osteosarcoma risk

**DOI:** 10.18632/oncotarget.26476

**Published:** 2018-12-21

**Authors:** Eleanor C. Semmes, Chenan Zhang, Kyle M. Walsh

**Affiliations:** Department of Neurosurgery, Duke University, Durham, NC, USA; Children’s Health and Discovery Institute, Department of Pediatrics, Duke University, Durham, NC, USA

**Keywords:** height, telomere length, osteosarcoma, intermediate phenotype, childhood cancer

Osteosarcoma predominantly affects adolescents and is the most common primary bone malignancy, yet its genetic etiology has not been fully elucidated. Cancer predisposition syndromes involving loss of tumor suppressor genes, such as Li-Fraumeni and hereditary retinoblastoma, are known to be associated with osteosarcoma [1]. Taller height and male sex have also been correlated with increased risk [2]. Genetic variants involved in DNA repair, height attainment, and telomere maintenance have been implicated in previous genetic epidemiology studies, but have not been identified in larger genome-wide association studies (GWAS) [1, 2].

There remain significant challenges to understanding the genetic etiology of relatively rare diseases, such as pediatric cancers, in the post-GWAS era. Despite being the most common malignancy of bone, there are only approximately 4 cases of osteosarcoma diagnosed per 1 million U.S. individuals each year according to SEER data [2]. The only osteosarcoma GWAS to date included 941 cases and identified two genome-wide significant risk loci at 6p21.3 and 2p25.2 [2]. However, these were not in regions previously hypothesized to play a role in osteosarcoma formation, nor have they been replicated in independent datasets. The power of GWAS to detect significant genetic contributions to pediatric malignancies will continue to be hampered by the limited number of cases available for study and the modest effect size of most individual risk alleles. Therefore, a more complete understanding of pediatric cancer heritability may necessitate new analytic approaches [3].

A solution to these challenges may lie in examining genetic variants associated with cancer risk factors, i.e. “intermediate phenotypes”, rather than variants directly associated with cancer [4]. This approach involves identifying putative risk factors from epidemiologic studies and determining variants associated with those phenotypes from previously published GWAS. Since GWAS of normal variation in traits like height attainment or telomere length can be conducted in generic cohorts constructed without regard to cancer status, they can attain large sample sizes and adequate statistical power to detect many important loci [5, 6]. Thus, examining the genetics of “intermediate phenotypes” may elucidate the complex genetic architecture of rare diseases and provide greater insights into the heritability and pathogenesis of malignancies such as childhood osteosarcoma [4].

A recent study by Zhang *et al.* investigated the role of genetic determinants of height on osteosarcoma risk using this approach, thereby overcoming some of the difficulties previously outlined [1]. The study included 864 cases and 1879 controls from the California Osteosarcoma Case-Control Study and National Cancer Institute/Geisinger Osteosarcoma Data Set. The authors used hundreds of previously-identified single nucleotide polymorphisms (SNPs) from GWAS of adult height, childhood height and birth length to assess the relationship between height genetics and osteosarcoma risk [1, 5]. They constructed polygenic scores of height to analyze the combined as well as individual effects of these trait-associated SNPs.

Polygenic scores for adult and childhood height were shown to independently contribute to increased osteosarcoma risk (*P* = 0.027 and *P* = 0.023 respectively). These results indicated that genetic variation corresponding to a 0.5 cm increase in childhood height attainment and a 1.7 cm increase in adult height attainment each independently confer 10% greater odds of osteosarcoma [1]. Additionally, Zhang *et al.* analyzed the effect of single and grouped SNPs within common biological pathways to understand mechanistically how height might contribute to tumorigenesis. A single SNP located in a regulatory region implicated in osteoblast regulation and cartilage formation, rs8103992, was significantly associated with osteosarcoma risk after adjustment for multiple comparisons. Grouped height-associated SNPs related to gastrin signaling were associated with osteosarcoma risk as well [1].

The use of polygenic scores, combining the additive effects of multiple known variants that contribute to specific phenotypes, has implications beyond osteosarcoma research. In another recent study, Walsh *et al.* created a polygenic score for longer telomere length and tested it for association with multiple childhood cancers. They observed strong evidence of association between longer telomere length and increased neuroblastoma risk, and statistically significant but more modest associations with osteosarcoma and acute lymphoblastic leukemia risks [7]. Harnessing knowledge from existing GWAS of “intermediate phenotypes” in this manner may allow for a more robust understanding of diseases for which GWAS have offered limited insight [3, 4].

Such approaches can help to combat the ‘missing heritability’ problem that has plagued human genetics research. The concept of ‘missing heritability’ has been well-characterized [3, 4] and refers to the limit of known genetic variants to account for a disease’s heritability based on population-level studies. This ‘missing heritability’ is multi-factorial and likely due to undetected rare/structural/epigenetic variants as well as poorly understood gene-gene and gene-environment interactions [3]. By examining “intermediate phenotypes” through analyses of individual SNPs and polygenic scores, it may be possible to uncover an additional source of this ‘missing heritability’ [4].

In summary, investigating the genetics of disease-associated traits such as height or telomere length is a promising approach to understanding complex disease etiology. This method will be most useful for future research involving rare diseases, for which GWAS remain underpowered.
